# The Interaction Between Non-Coding RNAs and Calcium Binding Proteins

**DOI:** 10.3389/fonc.2022.848376

**Published:** 2022-03-04

**Authors:** Soudeh Ghafouri-Fard, Jamal Majidpoor, Hamed Shoorei, Bashdar Mahmud Hussen, Hazha Hadayat Jamal, Aria Baniahmad, Mohammad Taheri, Majid Mokhtari

**Affiliations:** ^1^ Department of Medical Genetics, School of Medicine, Shahid Beheshti University of Medical Sciences, Tehran, Iran; ^2^ Department of Anatomy, Faculty of Medicine, Infectious Diseases Research Center, Gonabad University of Medical Sciences, Gonabad, Iran; ^3^ Department of Anatomical Sciences, Faculty of Medicine, Birjand University of Medical Sciences, Birjand, Iran; ^4^ Department of Pharmacognosy, College of Pharmacy, Hawler Medical University, Erbil, Iraq; ^5^ Center of Research and Strategic Studies, Lebanese French University, Erbil, Iraq; ^6^ Department of Biology, College of Education, Salahaddin University-Erbil, Erbil, Iraq; ^7^ Institute of Human Genetics, Jena University Hospital, Jena, Germany; ^8^ Urology and Nephrology Research Center, Shahid Beheshti University of Medical Sciences, Tehran, Iran; ^9^ Skull Base Research Center, Loghman Hakim Hospital, Shahid Beheshti University of Medical Sciences, Tehran, Iran

**Keywords:** non-coding RNA, calcium binding protein, lncRNA, circRNA, miRNA

## Abstract

Calcium binding proteins (CBP) are a group of proteins mediating the effects of calcium on cellular functions. These proteins can regulate calcium levels inside the cells and contribute in several cellular functions through transporting this ion across cell membranes or decoding related signals. Recent studies have shown that several non-coding RNAs interact with CBPs to affect their expression or activity. The interactions between these transcripts and CBPs have implications in the pathoetiology of human disorders, including both neoplastic and non-neoplastic conditions. In the current review, we describe the interactions between three classes of non-coding RNAs (long non-coding RNAs, circular RNAs, and microRNAs) and a number of CBPs, particularly CAB39, S100A1, S100A4, S100A7 and S100P. This kind of interaction has been verified in different pathological contexts such as drug-induced cardiotoxicity, osteoblasts cytotoxicity, acute lung injury, myocardial ischemia/reperfusion injury, proliferative diabetic retinopathy, glomerulonephritis, as well as a wide array of neoplastic conditions.

## Introduction

Calcium is an important second messenger in cells whose effects are largely dependent on a number of diverse proteins, being named as calcium binding proteins (CBP), accordingly. These proteins can bind this ion in their certain domains. CBPs can regulate calcium levels inside the cells and contribute in several cellular functions through transporting this ion across cell membranes or decoding related signals (1). Based on the presence of the structural EF-hand domain, intracellular CBPs can be classified into two main classes, i.e., those containing this domain and those lacking this domain. Parvalbumin, calmodulin, S100 proteins and calcineurin are examples of the former class, while calreticulin, calsequestrin, annexins, protein kinase C (PKC) and sinaptotagmin are examples of the latter (1). Extracellular CBP has six main classes, based on the presence of EF-hand, EGF-like, γ-carboxyl glutamic acid (GLA)-rich, cadherin, and calcium-dependent (C)-type lectin-like domains or calcium binding pockets of family C G-protein-coupled receptors (1). Extracellular CBPs are incessantly surrounded by a concentration of 10^−3^M calcium which contributes in the activation or stabilization of specific enzymes acting as protease, nuclease, or lipase. On the other hand, intracellular CBPs, which act as muscle contraction, respond to an upsurge in calcium concentrations from 10^−7^ to 10^−6^M (2). Recent studies have shown that several non-coding RNAs interact with CBPs to affect their expression or activity. The interactions between these transcripts and CBPs have implications in the pathoetiology of human disorders, including both neoplastic and non-neoplastic conditions. In the current review, we describe the interactions between three classes of non-coding RNAs (long non-coding RNAs (lncRNAs), circular RNAs [circRNAs) and microRNAs (miRNAs)] and CBPs.

## Non-Coding RNAs and CAB39

**Figure 1:**
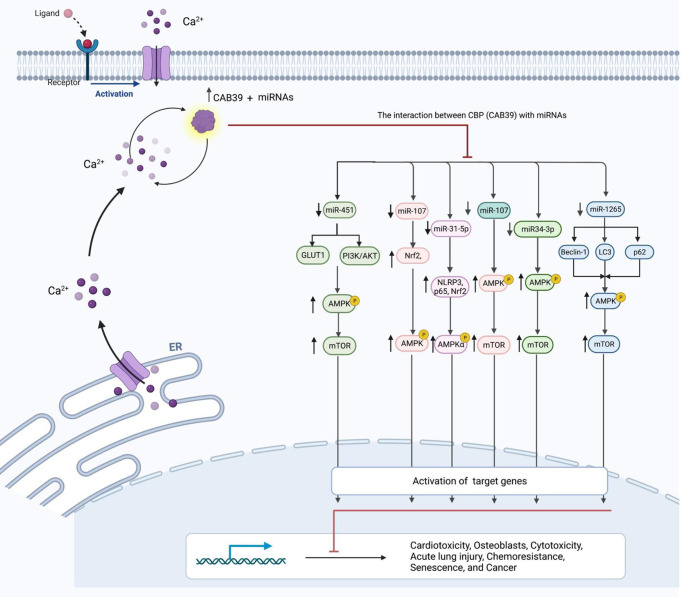
The connection between CAB39 and miRNAs, as well as their role in human diseases. Inhibition of miRNA has resulted in increased CAB39 levels and increased activity of AMPK pathway. Cab39 has therefore been found as a miRNAs target, and these miRNAs modulate cardiotoxicity, osteoblasts, cytotoxicity, acute lung damage, chemoresistance, senescence, and cancer development through this RNA.

Calcium-binding protein 39 (CAB39) is functionally associated with the Serine/Threonine Kinase STK11 and STRAD (3). This protein also promotes the construction of STK11/STRAD complexes and induces catalytic activity of STK11 (3). This protein has been found to affect the process of doxorubicin-induced cardiac injury. Experiments in an animal model of doxorubicin-induced cardiotoxicity have shown up-regulation of miR-451 levels. Suppression of miR-451 expression has reduced doxorubicin-associated whole-body wasting and cardiac atrophy, decreased heart damage, amended heart function, and enhanced contractile function of cardiomyocytes. Functionally, miR-451 suppression has led to enhancement of Cab39 levels and induced activity of AMPK signaling (**Figure 1**). Thus, Cab39 has been identified as the target of miR-451 through which this miRNA affects cardiac toxicity (4). Another study to find the mechanism of osteoblast cytoprotection has reported miR-107 as a CAB39-targeting miRNA. Functional experiments in OB-6 human osteoblastic cells have shown direct binding of this miRNA with CAB39 mRNA. Both wild-type miR-107 mimics and pre-miR-107-containing lentiviruses could inhibit CAB39 expression in osteoblasts. On the other hand, miR-107 antagonism could increase CAB39 expression, leading to activation of AMPK cascade. Suppression of miR-107 has significantly decreased dexamethasone-induced apoptosis in OB-6 cells and human osteoblasts. Moreover, antagomiR-107 could activate AMPK downstream Nrf2 cascade to suppress dexamethasone-associated oxidative injury (5). The interaction between miRNAs and CAB39 has also been implicated in the pathogenesis of acute lung injury. The CAB39-interacting miR-31-5p has been shown to be up-regulated in mice lung tissues upon injection of lipopolysaccharide. miR-31-5p silencing has relieved, while miR-31-5p mimic has aggravated lipopolysaccharide-induced inflammatory responses, oxidative injury, and pulmonary injury *in vivo* and *in vitro*. Functionally, miR-31-5p silencing has induced protective impact of AMPKα. In fact, Cab39 has an essential role in activation of AMPKα and protective effects of miR-31-5p antagomir (6). The interaction between miRNAs and CAB39 has an important role in the pathogenesis of cancers. For instance, miR-1265 can regulate cell proliferation and apoptosis in gastric cancer cells by targeting CAB39. miR-1265-mediated suppression of CAB39 interferes with oncogenic autophagy through modulation of AMPK/mTOR (7). On the other hand, miR-107-mediated suppression of CAB39 and subsequent activation of AMPK/mTOR signaling confers chemoresistance to colorectal cancer (8). **Table 1** summarizes the role of CAB39-interacting miRNAs in the pathogenesis of different disorders.

**Table 1:** Shows the interaction between CBP (CAB39) with miRNAs.

Disease	miRNA	Animal & Human Study	Cell Line	Target & *Pathway*	Conclusion	Ref
Cardiotoxicity	miR-451	C57BL/6 mice	H9c2	*AMPK/mTOR*	miR-451 silencing *via* activating CAB39 and AMPK could inhibit doxorubicin exposure-induced cardiotoxicity in mice.	(4)
Osteoblasts Cytotoxicity	miR-107	–	OB-6	Nrf2, *AMPK*	Inhibiting miR-107 *via* upregulating CAB39 and activating the AMPK-Nrf2 axis could act against oxidative injury and cytotoxicity induced by dexamethasone in osteoblasts.	(5)
Acute Lung Injury	miR-31-5p	C57BL/6 mice	alveolar macrophage cell (MH-S)	AMPKα, NLRP3, p65, Nrf2	miR-31-5p *via* inactivating CAB39/AMPKα axis could exacerbate lipopolysaccharide-induced acute lung injury.	(6)
Gastric Cancer (GC)	miR-1265	nude mice, 63 pairs of GC and adjacent normal samples	GES-1, MKN45, SGC7901, AGS, MGC803, HGC27, BGC823	Beclin-1, LC3, p62, *AMPK/mTOR*	miR-1265 by targeting CAB39 could regulate and apoptosis in GC and impair autophagy.	(7)
Colorectal Cancer (CRC)	miR-107	BALB/c nude mice	HCT-8, LoVo, 293T, HCT-116, HCT-116/L-OHP	*AMPK/mTOR*	miR-107 by targeting CAB39 could confer chemoresistance.	(8)
Lung Cancer	miR-451	–	H460, A549, LK2, HBE	GLUT1, *PI3K/AKT*	HPV16 E6/E7 *via* the PI3K/AKT pathway by relieving miR-451 inhibitory effect on CAB39 could promote glucose uptake of GLUT1 in lung cancer cells.	(9)
–	miR-34a-3p	–	dental pulp stem cells (DPSCs)	p53, p21, p16, *AMPK/mTOR*	Metformin-induced miR-34a-3p downregulation by targeting CAB39 *via* the AMPK/mTOR pathway could alleviate senescence in human DPSCs.	(10)

**Figure 2:**
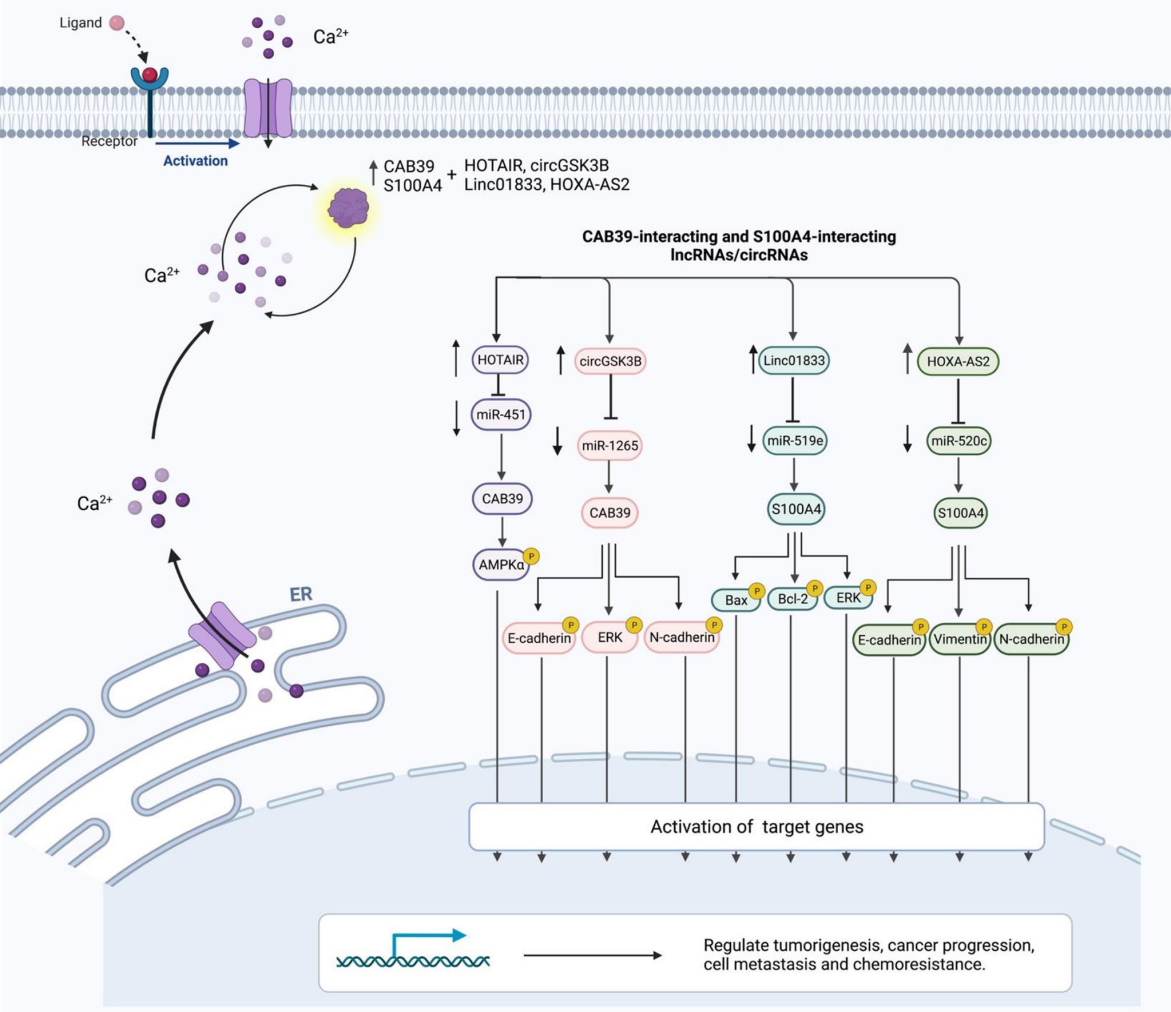
Interaction between CBPs (CAB39 and S100A4) and lncRNAs/circRNAs with their contribution in human disorders.

CircGSK3B (hsa_circ_0003763) is a circRNA that has indirect interaction with CAB39. This circRNA has been found to be up-regulated in hepatocellular cancer tissues and cell lines. In addition, expression levels of circGSK3B have been correlated with tumor bulk and vascular invasion. Functional studies have indicated the role of circGSK3B in the enhancement of proliferation, migratory potential, and invasiveness of hepatocellular carcinoma. Mechanistically, circGSK3B sponges miR-1265 to up-regulate expression of CAB39 (**Figure 2**). This circRNA has a role in reprogramming of glutamine metabolism. Taken together, circGSK3B/miR-1265/CAB39 axis has a role in enhancing proliferation, migration, invasion of this kind of cancer (11). HOTAIR is an example of lncRNAs that activates AMPKα *via* EZH2/miR-451/CAB39 axis regulation. CAB39 is involved in regulation of oxidative stress and cardiac myocyte apoptosis during ischemia/reperfusion injury (12). **Table 2** summarizes CAB39-interacting lncRNAs/circRNAs.

**Table 2:** CAB39-interacting lncRNAs/circRNAs.

Disease	lncRNA/circRNA	Animal & Human Study	Cell Line	Target &* Pathway*	Conclusion	Ref
Hepatocellular Carcinoma (HCC)	circGSK3B	–	Hep-G2, LO2, SMMC-7721, Hep3B, Huh7	miR-1265, E-cadherin, N-cadherin, Vimentin, *ERK*	Circular RNA circGSK3B by sponging miR-1265 and regulating CAB39 could promote cell proliferation, migration, and invasion in HCC.	(11)
Myocardial I/R Injury	HOTAIR	C57BL/6 mice	H9c2	AMPKα, BRD4, Bax, Bcl-2, SIRT1, MnSOD, Catalase, *AKT*	HOTAIR could activate AMPKα *via* regulating the EZH2/miR-451/CAB39 axis, therefore, it is involved in regulation of oxidative stress and cardiac myocyte apoptosis during I/R injury.	(12)

## Non-Coding RNAs and S100A4

**Figure 3:**
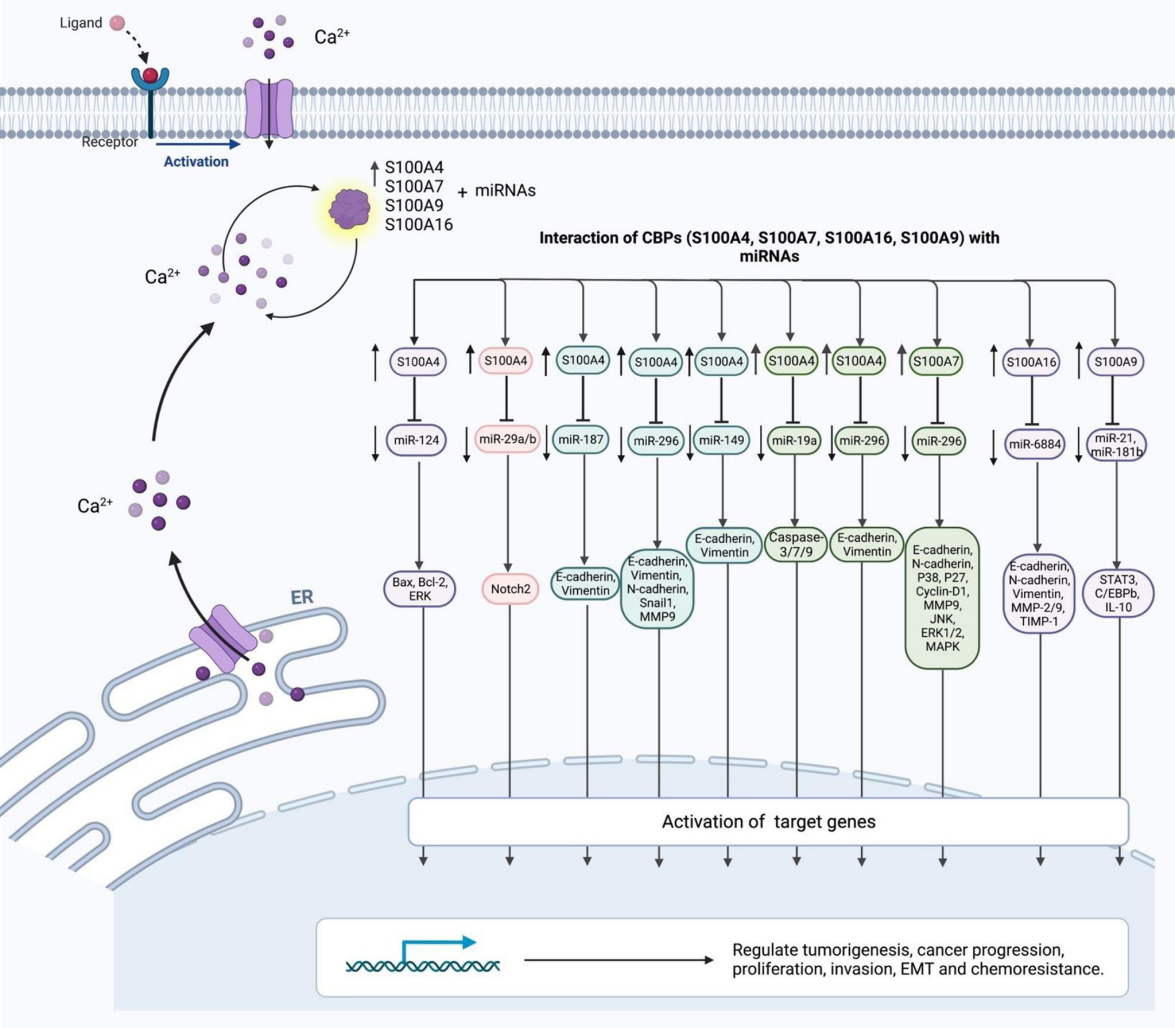
The interaction of CBPs (S100A4, S100A7, S100A16, S100A9) with miRNAs.

S100A4 is a member of the S100 CBP family, which is produced by tumor cells as well as stromal cells. S100 proteins are localized in the cytoplasm and/or nucleus of several kinds of cells and participate in the regulation of cell cycle transition and differentiation. The genes coding members of S100 family are clustered on chromosome 1q21 (13). This CBP has been shown to support tumorigenesis through stimulation of angiogenesis. A number of miRNAs have been shown to inhibit the expression of S100A4. For instance, miR-187-3p by targeting S100A4 could inhibit the metastasis and epithelial-mesenchymal transition (EMT) of hepatocellular carcinoma (14). Expression of S100A4 has been shown to be increased in ovarian cancer in association with clinical stage of these patients. Down-regulation of this CBP has reduced the mobility of ovarian cancer cells and their metastatic ability, while up-regulation of S100A4 has increased the invasive aptitude of these cells. miR-296 has been identified as an important upstream regulator of this CBP (**Figure 3**). Dysregulation of miR-296/S100A4 axis could facilitate EMT (15). Another study in bladder cancer has revealed that miR-149-3p could inhibit proliferation, migration, and invasion of malignant cells through targeting S100A4 (16). In colorectal cancer cells, miR-325-3p/S100A4 (17), miR-520c/S100A4 (18) and miR-296/S100A4 (19) have been identified as molecular axes that affect carcinogenesis. **Table 3** shows S100A4-interacting miRNAs.

**Table 3:** S100A4-interacting miRNAs.

Disease	miRNA	Animal & Human Study	Cell Line	Target & Pathway	Conclusion	Ref
Carotid Artery Balloon Injury	miR-124	SD rat	smooth muscle cell line A10	Bax, Bcl-2, ERK	miR-124 by targeting S100A4 could inhibit vascular smooth muscle cell proliferation.	(20)
Proliferative Diabetic Retinopathy (PDR)	miR-29a/b	–	HRMECs	Notch2, vascular endothelial cadherin	miR-29a/b cluster by targeting Notch2/S100A4 could suppress high glucose-induced EMT in human retinal microvascular endothelial cells.	(21)
Hepatocellular Carcinoma (HCC)	miR-187-3p	nude mice, 120 pairs of HCC and adjacent normal samples	MHCC97H, HepG2, SMMC7721, Huh7, Hep3B, LO2	E-cadherin, Vimentin, EMT	miR-187-3p by targeting S100A4 could inhibit the metastasis and EMT of HCC.	(14)
Ovarian Cancer (OC)	miR-296	Human tissue microarray (OV809)	SKOV-3, HO8910, HO8910-PM, OVCAR-3, Caov-3	E-cadherin, Vimentin, N-cadherin, Snail1, MMP9	By inducing EMT, the deregulated miR-296/S100A4 axis could promote tumor invasion in human OC.	(15)
Bladder Cancer	miR-149-3p	BALB/C nude mice	UM-UC-3	E-cadherin, Vimentin,	miR-149-3p by targeting S100A4 could inhibit proliferation, migration, and invasion of bladder cancer.	(16)
Anaplastic Thyroid Cancer (ATC)	miR-19a	–	FTC-133, 8505c, K1	Caspase-3/7/9	miR-19a could be involved in the progression and malignancy of ATC cells.	(22)
Colorectal Cancer (CRC)	miR-325-3p	–	Bone marrow, CT-26, 293T	–	miR-325-3p *via* targeting S100A4 could act as a regulator of osteoclastogenesis in osteolysis of CRC.	(17)
CRC	miR-520c	Mice, 59 pairs of CRC and adjacent normal samples	SW480, Rko, WiDr, DLD-1, HCT116, SW620, HT-29, Colo320DM, Caco-2, HCT-15	–	Epigenetic silencing of miR-520c could induce S100A4 and its mediated CRC progression.	(18)
CRC	miR-296	90 pairs of CRC and adjacent normal samples	HCT116, Caco-2, HT29, SW620, SW480, HIEC	E-cadherin, Vimentin,	miR-296 by targeting S100A4 could inhibit the metastasis and EMT of CRC.	(19)

A number of lncRNAs have been found to affect miRNA/S100A4 axes. These lncRNAs mainly act as molecular sponges for S100A4-interacting miRNAs, thus releasing S100A4 from inhibitory effects of these miRNAs. For instance, HOXA-AS2 through regulating miR-520c-3p/S100A4 (23) and miR-520c-3p/S100A4 (24) axes could affect pathogenesis of papillary thyroid cancer and acute myeloid leukemia, respectively. Moreover, Linc01833 *via* regulation of miR-519e-3p/S100A4 axis could enhance progression of lung cancer (25). **Table 4** shows S100A4-interacting lncRNAs.

**Table 4:** S100A4-interacting lncRNAs.

Disease	lncRNA	Animal & Human Study	Cell Line	Target & Pathway	Conclusion	Ref
Papillary Thyroid Cancer (PTC)	HOXA-AS2	BALB/c nude mice, 128 pairs of PTC, and adjacent normal samples	BHP5-16, TPC, K1, BHP2-7, Nthy-ori 3-1	miR-520c-3p, Vimentin, N-cadherin, E-cadherin	HOXA-AS2 by regulating miR-520c-3p/S100A4 pathway could promote PTC progression.	(23)
Acute Myeloid Leukemia (AML)	HOXA-AS2	nude mice, 48 AML patients	U937, THP-1, U937/ADR, THP-1/ADR	miR-520c-3p	Knockdown of HOXA-AS2 *via* the miR-520c-3p/S100A4 axis could suppress adriamycin-based chemotherapy resistance of AML.	(24)
Lung Adenocarcinoma (LUAD)	Linc01833	–	A549, HCC4006	miR-519e-3p, Vimentin, E-cadherin, Cytokeratin	Linc01833 *via* the miR-519e-3p/S100A4 axis could enhance LUAD progression.	(25)

## Non-Coding RNAs and S100A7

S100A7 is another member of the S100 family of proteins which contains 2 EF-hand domains. S100A7 differs from the other members of this family in the absence of calcium binding capacity in one of its EF-hand domains, which is located at the N-terminus. S100A7 has been shown to regulate metastatic ability of ovarian cancer cells and chemoresistance phenotype through modulation of MAPK pathway (**Figure 3**). This CBP has been shown to be targeted by miR-330-5p (26). Moreover, S100A7 has been found to be a direct target of miR-26b-5p. In fact, miR-26b-5p can suppress proliferation, migration and invasiveness of intrahepatic cholangiocarcinoma cells through deceasing expression of S100A7 (27). **Table 5** shows S100A7-interacting miRNAs.

**Table 5:** S100A7-interacting miRNAs.

Disease	miRNA	Animal & Human Study	Cell Line	Target & Pathway	Conclusion	Ref
Ovarian Cancer (OC)	miR-330-5p	40 EOC tissues and 10 normal epithelial ovarian tissues	Caov3, SKOV3, Caov3/Cis, SKOV3/Cis	E-cadherin, N-cadherin, P38, P27, Cyclin-D1, MMP9, JNK, *ERK1/2, MAPK*	S100A7 *via* MAPK signaling could regulate OC cell metastasis and chemoresistance.	(26)
Intrahepatic Cholangiocarcinoma (ICC)	miR−26b−5p	20 pairs of ICC and adjacent normal samples	RBE, HCCC-9810	–	miR−26b−5p by targeting S100A7 could regulate cell proliferation, invasion, and metastasis in human ICC.	(27)

## Non-Coding RNAs and S100A1

Similar to the majority of S100 proteins, binding of S100A1 with calcium results in great conformational alterations which facilitate interaction of this CBP with several protein targets. Targets of this CBP are those participating in calcium-related signal transduction, neurotransmitter release, cytoskeletal and filament associated proteins, transcription factors, a number of different proteins with enzymatic functions, and other CBPs, particularly S100B, S100A4 and S100P (28). Suppression of S100A1 expression has been suggested as a therapeutic modality for treatment of various disorders such as neurological disorders, diabetes mellitus, heart failure, and numerous kinds of malignancies (28). FOXD2-AS1 is the only lncRNA whose interactions with S100A1 have been verified. This lncRNA through modulation of S100A1/Hippo (29) and miR-363-5p/S100A1 pathways (30) can participate in the pathogenesis of breast cancer and nasopharyngeal carcinoma, respectively (**Table 6**).

**Table 6:** S100A1-interacting lncRNAs.

Disease	lncRNA	Animal & Human Study	Cell Line	Target & *Pathway*	Conclusion	Ref
Breast Cancer (BCa)	FOXD2-AS1	BALB/c nude mice, Dataset	MCF-10A, MCF-7, BT-549, MDA-MB-468, MDA-MB-453	Cyclin-E1, CDK2, p21, MMP2/9, YAD, LATS1, MST1/2, *Hippo*	FOXD2-AS1 *via* the S100A1/Hippo signaling pathway could regulate the tumorigenesis and progression of BCa.	(29)
Nasopharyngeal Carcinoma (NPC)	FOXD2-AS1	BALB/c nude mice, 50 pairs of NPC and adjacent normal samples	SUNE-1, CNE-1-2, HNE-1, C666-1, HONE-1	miR-363-5p	FOXD2-AS1, by modulating miR-363-5p/S100A1 pathway, could participate in NPC carcinogenesis.	(30)

## Non-Coding RNAs and S100P

S100P is another member of S100 CBPs that mediate calcium-dependent signal transduction (31). S100P has been primarily isolated from the placenta (32). From an evolutionary point of view, S100P is regarded as a novel gene, existing only in the vertebrate genomes (33). As S100P is expressed in the uterus during the rhythmic hormonal changes, it might be associated with embryonic implantation/development (33). Yet, the role of S100P has been mostly investigated in the context of cancer (33). Two independent studies have assessed association between S100P and non-coding RNAs in pancreatic cancer. First, miR-495 has been shown to suppress pancreatic carcinogenesis by targeting S100P (34). Secondly, circ_0092314 has been shown to induce EMT in this type of cancer through sponging miR-671 and releasing S100P from its inhibitory effects (35). **Table 7** shows S100P-ineracting miRNAs and circRNAs.

**Table 7:** S100P-ineracting miRNAs and circRNAs.

Disease	miRNA/circRNA	Animal & Human Study	Cell Line	Target & Pathway	Conclusion	Ref
Pancreatic Cancer	miR-495	GEO dataset	HPDE6c7, 293T, Sw1990, Bxpc-3	–	miR-495 by targeting S100P could perform suppressive roles in pancreatic adenocarcinoma.	(34)
Pancreatic Cancer	circ_0092314	Nude mice, PAAD tissues	AsPC-1, BxPC-3, SW-1990, PaCa-2, HPDE6-C7	miR-671, E-cadherin, Vimentin, *AKT*	Circ_0092314 *via* elevating S100P expression by sponging miR-671 could induce EMT.	(35)

## Non-Coding RNAs and Other Calcium Binding Proteins

S100A8, S100A9, S100A10, S100A11, S100A14, S100A16, NECAB3 and SMOC2 are other CBPs whose interactions with non-coding RNAs have been verified in the context of human disorders (**Tables 8** and **9**). LINC00174 *via* targeting regulates miR-320/S100A10 axis could increase malignant phenotypes (40). SNHG8 is another lncRNA which serves as a sponge for miR-1270 to up-regulate expression of S100A11 and promote progression of ovarian cancer (41). In the context of lung cancer, CASC9 has been found to sponge miR-335-3p and induce expression of S100A14 (42). In addition, GNAS-AS1 serves as a sponge for miR-4319 to increase expression of NECAB3 and regulate macrophage polarization (43).

**Table 8:** miRNAs interacting with other calcium binding proteins.

Disease	miRNA	Calcium Binding Pr.	Animal & Human Study	Cell Line	Target & Pathway	Conclusion	Ref
Endometrial Carcinoma (EC)	miR-24	S100A8	46 pairs of EC and adjacent normal samples	HEC-1A, 293T, HEC-1A/Pax	–	miR-24 by targeted silencing of the S100A8 gene could act as a tumor-suppressing gene and increase chemotherapy sensitivity of EC cells to paclitaxel.	(36)
Chronic Sepsis	miR-21, miR-181b	S100A9	C57BL/6N S100a9 knockout mice	Gr1+CD11b+	STAT3, C/EBPb, IL-10	S100A9 by inducing both miR-181b and miR-21 could maintain myeloid-derived suppressor cells in chronic sepsis.	(37)
Gastric Cancer (GC)	miR-6884-5p	S100A16	30 pairs of GC and adjacent normal samples	AGS, MKN45, BGC-823, SGC-7901, MGC-803, FTE187	E-cadherin, N-cadherin, Vimentin, MMP-2/9, TIMP-1	miR-6884-5p by targeting S100A16 could regulate proliferation, invasion, and EMT of GC cells.	(38)
Glomerulonephritis	miR-17-5p	SMOC2	–	AB8/13	NF-κB, TGFβ1, Fibronectin-1, Collagen-I/II, α-SMA, SMAD-2/3	miR-17-5p by suppressing SMOC2 *via* the NF-κB and TGFβ signaling could restrain the dysfunction of Ang-II induced podocytes.	(39)

**Table 9:** lncRNAs interacting with other calcium binding-proteins.

Disease	lncRNA	Calcium Binding Pr.	Animal & Human Study	Cell Line	Target & *Pathway*	Conclusion	Ref
Hepatocellular Carcinoma (HCC)	LINC00174	S100A10	45 pairs of HCC and adjacent normal samples	Hep3B, Huh7, SMMC-7721, L02	miR-320	LINC00174 *via* targeting regulates miR-320/S100A10 axis could increase malignant phenotypes.	(40)
Ovarian Cancer (OC)	SNHG8	S100A11	Mice, 19 pairs of OC and adjacent normal samples	IOSE, A2780, HOSE 11-12, SKOV3, HO8910, OVCAR3	miR-1270	SNHG8 *via* serving as a sponge for miR-1270 to regulate S100A11 could promote OC progression.	(41)
Non-small cell lung cancer (NSCLC)	CASC9	S100A14	43 pairs of NSCLC and adjacent normal samples	A549, H1299, BEAS-2B	miR-335-3p, MMP-2/9, N-cadherin, E-cadherin	Upregulation of CASC9 *via* inhibiting miR-335-3p and activating S100A14 could contribute to the progression of NSCLC.	(42)
NSCLC	GNAS-AS1	NECAB3	50 pairs of NSCLC and adjacent normal samples	PC9, SPCA1, H358, A549, H1299, 16HBE	miR-4319, IL-10, Arg-1	GNAS-AS1/miR-4319/NECAB3 axis by altering macrophage polarization could promote migration and invasion of NSCLC cells.	(43)

## Discussion

The interactions between ncRNAs and CBPs have been assessed in different contexts. Most of studies have been conducted in the context of cancer, where CBPs affect malignant features through a variety of mechanisms, particularly induction of EMT. CAB39 is among the mostly assessed CBPs in this regard. Notably, the functional effect of CAB39-interacting miRNAs on the cells is largely mediated through modulation of activity of AMPK/mTOR. S100A4 as another CBP has been shown to affect expression of EMT-markers such as E-cadherin, Vimentin, N-cadherin and Snail1. A number of miRNAs such as miR-187-3p, miR-296, miR-149-3p, miR-19a, miR-325-3p, miR-520c and miR-296 have been shown to affect carcinogenesis through modulation of expression of S100A4. Thus, S100A4-interacting non-coding RNAs are putative targets for design of novel therapeutic options against tumor metastasis and EMT. S100P and S100A16 are other CBPs whose interactions with non-coding RNAs are implicated in the process of EMT. In fact, miRNAs that affect expression of CBPs have been shown to bind with 3’ UTR of mRNAs coding for CBPs.

circRNAs and lncRNAs that affect expression of CBPs mainly act as molecular sponges for miRNAs. For instance, circGSK3B/miR-1265/CAB39, circ_0092314/miR-671/S100P, HOXA-AS2/miR-520c-3p/S100A4, HOXA-AS2/miR-520c-3p/S100A4, Linc01833/miR-519e-3p/S100A4, LINC00174/miR-320/S100A10, SNHG8/miR-1270/S100A11, CASC9/miR-335-3p/S100A14 and GNAS-AS1/miR-4319/NECAB3 are examples of these regulatory axes which are involved in the pathoetiology of human disorders, particularly cancers.

The regulatory effects of some miRNAs on their specific CBPs have been verified in different contexts. For instance, the inhibitory impact of miR-451 on CAB39 has been shown to be implicated in drug-associated cardiac toxicity as well as lung cancer. Similarly, CAB39 has been found as a target of miR-107 in both osteoblasts and colorectal cancer cells. Finally, S100A4 has been shown to be targeted by miR-296 in both ovarian and colorectal cancer cells. For other miRNAs, regulatory effects have been confirmed only in a single context.

Taken together, several miRNAs, lncRNAs and circRNAs can regulate expressions of CBPs and participate in the etiology of human disorders *via* this route. Identification of this type of interactions has practical significance in design of disorders which are associated with abnormal calcium signal transduction. Research in this field is still in its infancy and the functional associations between non-coding RNAs and several members of CBP family need to be clarified.

## Author Contributions

SG-F wrote the draft and revised it. MT and AB designed and supervised the study. HS, JM, BH, and HH collected the data and designed the figures and tables. All the authors read and approved the submitted version.

## Conflict of Interest

The authors declare that the research was conducted in the absence of any commercial or financial relationships that could be construed as a potential conflict of interest.

## Publisher’s Note

All claims expressed in this article are solely those of the authors and do not necessarily represent those of their affiliated organizations, or those of the publisher, the editors and the reviewers. Any product that may be evaluated in this article, or claim that may be made by its manufacturer, is not guaranteed or endorsed by the publisher.
